# The Costs of Austerity: Labor Emigration and the Rise of Radical Right Politics in Central and Eastern Europe

**DOI:** 10.3389/fsoc.2019.00069

**Published:** 2019-10-11

**Authors:** Maria Sigridur Finnsdottir

**Affiliations:** Department of Sociology, University of Toronto, Toronto, ON, Canada

**Keywords:** radical right, austerity, emigration, Europe, nationalism, labor

## Abstract

The right to free movement, to move and work freely within the European Union (EU), is a cornerstone right held by EU and European Free Trade Area (EFTA) citizens. Labor flows across the EU are, however, not uniform but are characterized by significant geographic disparities. In particular, since the 2008 global financial crisis, labor emigration, measured by the rate of EU citizens living and working in other EU countries, has increased exclusively among certain Central and Eastern European nations. This paper seeks to examine the link between labor migration, austerity policies, and the rise of radical nationalist politics. This paper uses Boolean analysis to examine the relationships between labor migration, nationalism, welfare support, and austerity. I argue that austerity measures have pushed certain Central and Eastern European countries into the roles of labor-sending nations, so that emigration and scarcity put pressure on traditional conceptions of belonging, fueling radical politics. In this way, austerity provides the material and ideological conditions under which emigration comes to be seen as a threat to the well-being of the nation, stoking support for nationalist populist parties.

## Introduction

The rise of the radical right, and of nationalist politics more generally, poses a pressing problem to democratic politics. Scholars working on the rise of the radical right in post-financial crisis Europe have pointed to the indirect role played by austerity in this phenomenon, underlining the relationship between economic grievances and support for antidemocratic politics (Lamprianou and Ellinas, 2017). The recent rise of radical right parties is all the more concerning in Central and Eastern Europe (CEE), where backsliding into antidemocratic politics has occurred especially quickly. This paper seeks to understand the effects of austerity on the rise of nationalist politics in the CEE countries by focusing on the role of labor emigration in driving perceptions of hardship and scarcity.

Hungary, Poland, Romania, Bulgaria, Croatia, Czechia, Slovakia, and Slovenia are of particular interest here. While their level of involvement with the Soviet Union varied, all these countries share a history of communist authoritarian rule. Of these, labor emigration has increased in the past decade in Bulgaria, Hungary, Slovakia, Poland, and Romania (Eurostat, 2018). Clearly then, while freedom of movement is one of the core rights held by all European Union (EU) and European Free Trade Area (EFTA) citizens, there are significant geographic disparities in emigration rates. These patterns in inter-EU migration suggest that certain CEE nations have transitioned into the role of informal labor-sending states. Importantly, labor-sending nations are not only active participants in migration systems but are also transformed by emigration. Beyond the economic effects of mass migration on labor-sending nations, the pressure put on understandings of national identity by emigration has profound effects on the politics of the sending nation (Lee, 2017). This is especially the case in contexts of economic hardship, when emigration is understood as a threat to the well-being of the nation.

Nationalist and exclusionary politics build on popular anxieties about national outsiders, as well as their potential effect on the nation. As a consequence, there is a large body of research focusing on the effects of immigration on the demand for radical right politics. However, the literature shows that the relationship between migration patterns and the rise of radical right politics is not yet clear, speaking to the need to better understand the dynamics of nationalism within both labor-sending and labor-receiving contexts. Thus, this paper seeks to understand how, within the context of austerity politics, labor emigration triggers perceptions of scarcity and provides a rich context for nationalist politics. Specifically, I argue that austerity policies bring about the material and ideological circumstances under which emigration comes to be perceived as a threat, in turn spurring support for radical right politics. I will begin with an overview of labor migration within the EU, before moving on to a discussion of European austerity and nationalist politics, as well as a more specific examination of the rise of radical right politics in the CEE countries. I then present my analysis, consisting primarily of Boolean analysis presented in a truth table. I conclude with a discussion of the relationship between increasing emigration and support for radical right and nationalist politics.

## Labor Migration Within the EU

One of the fundamental rights granted to citizens of the EU, and of the EFTA, is the right to move and work freely throughout the EU and EFTA (Eurostat, 2018). For this reason, inter-EU migrants represent a special category of migration—because of their EU/EFTA citizenship status, they are not reliant on visas to migrate and cannot easily be denied the right to work (Waterbury, 2018). According to (Eurostat, 2018), in 2017, at least five out of every 100 EU/EFTA citizens were residing in another EU/EFTA nation than the one they were born in, an increase from 2007 when it was about 3.5% of the EU/EFTA population. However, there are significant geographic disparities in rates of emigration; specifically, the postcrash years have seen an increase in emigration isolated to a few Eastern European nations.

**Figure 1:**
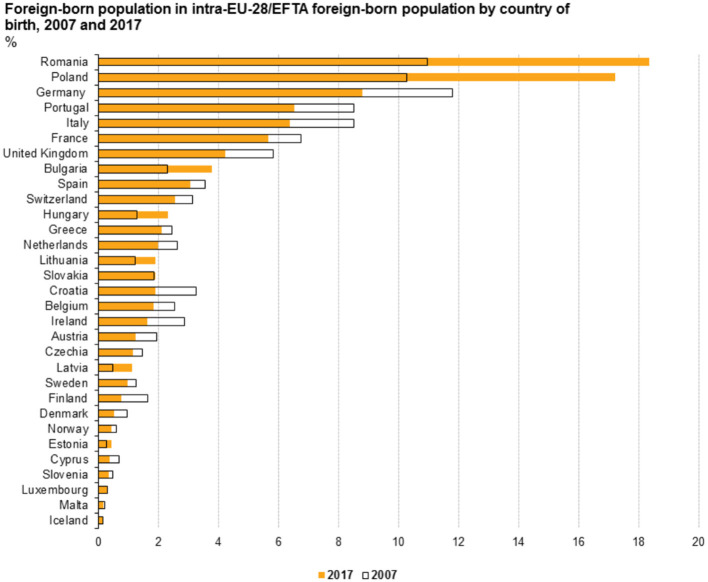
Inter-European Union (EU)/European Free Trade Area (EFTA) migration (Eurostat, 2018).

Figure 1 (Eurostat, 2018) shows the proportion of EU/EFTA citizens who were living and working in a different EU/EFTA nation in 2007 and 2017. This rate increased over the last decade exclusively in Romania, Poland, Bulgaria, Hungary, Lithuania, Latvia, and Estonia. In fact, as the graph shows, almost one-fifth of those born in Romania were, in 2017, residing in another EU/EFTA country (Eurostat, 2018). Similarly, about 17% of Polish-born EU citizens were found to be residing in other EU/EFTA nations. While the percentages were much lower in the other five nations than in Romania and Poland, they nonetheless represent significant increases over the past decade (Eurostat, 2018).

Data collected by the (Eurostat, 2019a) further show that, in 2017, emigrants outnumbered immigrants in Bulgaria, Croatia, Latvia, Lithuania, Poland, and Romania. Beyond that, in 2018, the majority of immigrants in Belgium, Ireland, Cyprus, Luxembourg, Malta, the Netherlands, Austria, Romania, Slovakia, and the United Kingdom were citizens of another EU nation, meaning that inter-EU migration was responsible for most of the immigration in those countries (Eurostat, 2019a). At the same time, the proportion of the population made up by non-nationals remains low across Eastern Europe; in Lithuania, Romania, and Poland, non-nationals represent <2% of the population (Eurostat, 2019a,b). These trends of increasing emigration and low immigration have had a significant impact on the national population in several Eastern European countries: Bulgaria, Croatia, Hungary, Romania, Latvia, Lithuania, and Poland all experienced a population decrease between 2007 and 2018 (Eurostat, 2019b; Rice-Oxley and Ranking, 2019). Given this context, it is perhaps unsurprising that recent polls have shown that many nations in Southern and Eastern Europe now express more worry about emigration than immigration, including Romania, Hungary, and Poland (Rice-Oxley and Ranking, 2019).

In sum, one of the most significant trends in Europe since the 2008 financial crisis has been the movement of people from east to west. However, this emigration is all the more interesting in that it appears, at least for now, to consist mostly of the permanent relocation of educated young people (Waterbury, 2018). Some scholars have viewed this east-to-west migration as an indictment of the corruption and ineptitude of many Central and Eastern European governments; in fact, according to World Bank rankings, the Soviet successor states remain among the most corrupt in the world (Holmes, 2013; Waterbury, 2018). However, it has also been widely attributed to a lack of economic opportunity in the sending nations (Waterbury, 2018). In line with general theories of migration, which emphasize the push–pull dynamics driving migration, differences between European countries in quality of life likely play a significant role in driving migration (Lee, 2017). This dynamic is seen clearly in the case of Romania, where emigration has increased drastically over the past decade and where about one-third of households are at risk of poverty and social exclusion (Dupré et al., 2014; Eurostat, 2018).

Austerity has also been linked to increasing emigration from CEE countries. While the post-2008 spike in emigration was prompted, to a large degree, by the contraction of the economy and the increase in unemployment caused by the financial crisis, the austerity measures put into place afterwards, which weakened social benefits and protections, also significantly contributed to the outward movement of labor from many Baltic and Eastern European nations (Juska and Woolfson, 2015). Some scholars explain this phenomenon as the result of the labor market segmentation driven by austerity, where the primary sector of skilled employment remains relatively secure, while the secondary sector of low-skill work is increasingly deregulated, becoming more precarious, and driving people to look for alternatives in other places (Juska and Woolfson, 2015). At its peak, austerity pushed emigration to such levels that national populations across the Baltics actually decreased (Juska and Woolfson, 2015; Eurostat, 2019b).

Unlike most of Southern Europe, many of the CEE nations willingly undertook austerity reforms during the recession. The Southern European countries, most notably Greece, Italy, Portugal, and Spain, were heavily impacted by the downturn in markets following the 2008 financial collapse, and the subsequent austerity measures imposed by the EU and the International Monetary Fund (IMF) were highly controversial (Koukiadaki et al., 2016). In Greece, in particular, austerity has been tied to increasing political unrest and polarization (Walter, 2016). Conversely, of the eight CEE countries studied here, only Hungary and Romania received bailout packages from the IMF, while Slovenia and Slovakia, as parts of the Eurozone, implemented austerity measures in accordance with European Monetary Union guidelines (Santa, 2012; Cankar and Petkovsek, 2013; Walter, 2016).

Following the 2008 market collapse, Hungary, already carrying a significant amount of state debt, was the first EU country to turn to the IMF for help in financing the government (Gyorffy, 2015). In exchange for bailout funds, the Hungarian government was required to put in place strict spending cuts, including freezing public sector wages, raising the retirement age, and slashing welfare program budgets (Gyorffy, 2015). Romania, for its part, instituted some of the most severe austerity reforms in Europe under the supervision of the IMF after the 2008 crash (Stoiciu, 2012; Lupu, 2014).

Fiscal reforms that introduce expenditure cuts tend to be politically unpopular and thus treacherous for those politicians pushing them through (Gyorffy, 2015). Nonetheless, several CEE nations—including Bulgaria, Croatia, Czechia, and Poland—undertook austerity programs without external pressure. Poland, interestingly, implemented spending cuts and required local governments to balance their budgets despite being the only EU nation to have avoided a recession in the years after the 2008 market collapse (Rae, 2012). The IMF was also not involved in the austerity measures—including cuts to infrastructure and tax increases—put through in Croatia (Sonje, 2012). Czechia implemented some of its most severe austerity measures before the recession even hit the country: neoliberal reforms undertaken under the leadership of social democrats (Cisar, 2017).

A significant body of work exists that examines the experiences of European migrants in their receiving countries, focused particularly on the social and political integration of Eastern European migrants in Western Europe, as well as the discrimination faced by these migrants in Western Europe. Even well-educated Eastern Europeans often end up in low-skilled service work once in Western Europe; nonetheless, Eastern Europeans are generally identified as economic migrants, although the factual basis of this assumption is disputed (Parutis, 2014; Gilmartin and Migge, 2015). Furthermore, many Eastern European migrants face significant barriers to social and political integration, including linguistic and cultural differences and various forms of discrimination, both in their workplaces and in their broader social spheres (Gilmartin and Migge, 2015).

The trend of east-to-west migration points to a specific kind of inter-EU migration system, where CEE countries are taking on the role of labor-sending countries. Given the freedom of movement guaranteed to EU/EFTA citizens, these more recent waves of migration pose a particular problem for the labor-sending nations. On a more bureaucratic level, the power of the state to manage emigration through trade agreements and visas has been largely replaced by informal family networks in shaping migration patterns within the continent (Waterbury, 2018). Furthermore, emigrants are overall more difficult to track than immigrants as individuals generally have more need of the state in the receiving country than in the sending one (Eurostat, 2019a). This has the added effect of making migration difficult to track on the part of the state; this is especially the case for circular or temporary migrants, who may not be accurately counted in their residency (Waterbury, 2018).

On top of these bureaucratic issues, the heterogeneous nature of this east-to-west migration means that it is significantly weaker as a symbolic resource when compared to historical cross-border kin communities (Waterbury, 2018). In the post-Iron Curtain countries, this is taking place in a larger context of tension over the ethnic and symbolic boundaries of national belonging, complicated by high levels of emigration (Waterbury, 2018). According to Waterbury (2018), this is further reflected in the political integration of recent emigrants—specifically in their voting rights—where certain states, such as Hungary and Bulgaria, have severely restricted non-resident citizens' voting rights in their privileging of the historical kin community of the nation. Thus, mass emigration, and the emergence of trans-territorial communities, puts pressure on definitions of national identity (Lee, 2017). Thus, just as emigrants are fundamentally impacted by their emigration experience, labor-sending nations are also transformed by mass emigration.

Scholars working on more traditional labor-sending states, such as Mexico and the Philippines, have looked not only at the ways in which these states work to regulate and control the migration of their citizens but also at how migrants experience both sending and receiving societies (Rodriguez, 1999; Lee, 2017). Of particular interest here is how mass migration has been shown to reshape national identities; for example, state-brokered labor emigration has birthed new national identities in the Philippines, ones where working overseas is seen as fulfilling a civic duty (Rodriguez, 2010). However, more works needs to be done on how nationalisms are impacted by free movement of labor, as well as how states and national politics are transformed by the emergences of these new nationalisms.

## Austerity and Nationalism in Postcrash Europe

In its most general sense, nationalism refers to the process of building a social–political identity from which a national society can be constructed (Breton, 1988). Breton (1988) argues that nationalism, as an ideology, has four key components: principles of inclusion or exclusion; a conception of the “national interest”; comparisons with other groups; and specific views as to the ways in which the social environment can threaten or support the group (Breton, 1988). While national identity can be drawn up around civic principles (civic nationalism), it can also be based on a more explicitly ethnocentric basis, where the society is based on cultural unity, and the basis for inclusion within that society is ethnic (Fozdar and Low, 2015). That said, the exclusionary nature of the national identity being constructed depends, in large part, on the criteria for inclusion and exclusion, as well as on the discursive framing of the “Other.”

While there is significant diversity in the specific policies of radical right parties, this new field of politics is united by two key characteristics: a xenophobic ethnonationalism and an antiestablishment populism (Rydgren, 2007). Radical right parties also generally embrace a policy of monism and advocate strengthening the nation by making it more ethnically homogeneous and returning society to more “traditional” values (Rydgren, 2007). Beyond that, Rydgren (2007) argues that their platforms tend to be embedded in a sort of sociocultural authoritarianism, one that places the good of the collective above that of the individual. This is generally manifested in a distrust in global elites, as well as of immigrants (Rydgren, 2007). Given this xenophobic ethnonationalism, it follows that the radical right works to tie ethnicity to nationality, both in rhetoric and in policy, so that the “Other” is also understood ethnically and nationally.

However, nationalist politics are not restricted to the most extreme ends of the political spectrum. Even in mainstream politics, nationalism can be seen in the privileging of an ethnic understanding of citizenship (*jus sanguinis*) over a territorial one (*just soli*) (Rydgren, 2007). Similarly, the reinvigorated insistence on language and cultural proficiency tests as a requirement of citizenship demonstrates a tendency toward ethnonationalist conception of citizenship across the West: of the 27 EU member states, only six had citizenship/language tests in 2006, compared to 18 in 2013 (Wodak, 2013).

Within this context, support for radical right parties has been growing across significant portions of the Western world, including across Europe, especially in the last five or so years (Roodujin, 2015). In Hungary, Jobbik has garnered a significant share of the votes in recent elections. The Alternative for Germany (AfD) has come to play a powerful part on the national political stage in Germany, as has Golden Dawn in Greece (The New York Times, 2016). The Rassemblement National (previously known as the Front National) in France, despite not holding many seats, had a presidential candidate make it into the last electoral round in the 2017 election (Kirk and Scott, 2017). Even the Scandinavian nations, known for their progressive politics, have strong radical right parties involved in local and national politics (Coman, 2015). The 2019 EU parliament elections contributed to an even more fragmented and polarized politics, as groups on the extremes of both ends of the political spectrum made gains; in particular, nationalists in France, Italy, and the United Kingdom did better than expected in the elections, as did Greens in German and Ireland (Erlanger and Specia, 2019). In view of this trend, a significant amount of work has been done in an attempt to understand the causes for both radical right party success and electoral support.

In this vein, some researchers have turned their eye to the more structural causes behind the rise of the radical right. Given the focus within nationalist party rhetoric on immigration, this literature has grappled with understanding the complex relationships between immigration, unemployment, and radical right support. Specifically, scholars have looked at the role of high immigration in the context of high unemployment and economic hardship as key to creating a fertile ground for radical right politics (Golder, 2003; Rydgren, 2007, 2008; Halla et al., 2017). For one, Cochrane and Nevitte (2014) show that unemployment rates only correlate with anti-immigrant sentiment in the context where there is already a radical right political presence, arguing that these factors only matter when there is already a group working to rhetorically tie immigration to unemployment, thereby framing immigrants as a threat to the well-being of the “people.” Within the open-border context of the EU, this concern with immigration—and, more generally, with the movement of peoples across borders—is often translated into Euroscepticism, shedding light on the popularity of anti-EU politics on the radical right (Kentmen-Cin and Erisen, 2017).

One of the defining characteristics of radical right politics is an exclusionary conception of national identity—an ethnonationalist conception of national belonging (Rydgren, 2007). Given the importance of territory to the understanding of nation prevalent in Eastern Europe, the vast emigration of ethnic nationals looking for better economic opportunity is perceived as a threat to the nation. Put simply, the exclusionary practices directed at national outsiders have a flip side, one that is important to understand in contexts of high emigration and low immigration. The movement of national insiders—to whom access to the state is restricted—out of the country disrupts the tight links between state and citizen in exclusionary regimes, thereby disrupting exclusionary conceptions of national identity. This raises the possibility that the move to radical right politics is, at least in part, prompted by a desire to reinforce traditional, territorially bounded, understandings of national belonging.

Work on the radical right in the years since the 2008 financial crisis has found evidence for a link between austerity measures and support for radical and nationalist politics, albeit indirect. In practice, austerity measures mean a decrease in social spending, weakening of the social protection mechanisms of the state, and the deregulation and restructuring of capital–labor relations (Carastathis, 2015). Beyond these concrete measures, austerity also refers to a rhetoric of fiscal severity, at both the individual and state levels, as the only morally responsible choice when faced with economic recession and crisis (Carastathis, 2015). The postcrash years saw austerity measures put into effect across Europe in an effort to decrease deficits and declines, meaning widespread cutbacks to public services, including unemployment benefits, public sector wages, and pensions (Pietras, 2009). Many politicians, bureaucrats, and economists believed that the implementation of austerity measures would pay off in the long run, despite the short-term hardships caused; however, more recent work has suggested that the austerity measures put in place after the financial crisis have caused long-lasting damage to the European economy (Gyorffy, 2015; Gechert et al., 2018). However, economic grievances do not appear to directly predict radical right voting; rather such grievances erode public trust in political institutions, leading voters to look for alternatives such as radical right parties (Lamprianou and Ellinas, 2017). Importantly, institutional legitimacy has been shown to be a stronger negative predictor of anti-immigrant sentiment in Eastern Europe than in Western Europe (Ceobanu and Escandell, 2008).

A second connection between austerity and radical right support is concern over the scarcity of state resources. Concerns with the scarcity of state resources are in turn often accompanied by concerns over the supposed misuse of state resources by outsiders—a kind of rhetoric that has also been referred to as welfare chauvinism. Welfare chauvinism is defined as “a system of collective social protection that is restricted to those belonging to the ethnically defined community who has contributed to it” (Careja et al., 2016, p. 436). That is, welfare chauvinism arises when national outsiders are perceived to be taking more of the state's resources than they are entitled to and, in this way, as posing a threat to the economic and social well-being of nationals (Hjorth, 2016). Austerity, and the austerity-driven perceptions of scarcity, then can drive welfare chauvinism to the forefront of politics, increasing the political salience of anti-immigrant politics.

The austerity-driven cuts to welfare systems across Europe, thus, contribute to welfare chauvinist attitudes, as well as the attendant anti-immigrant sentiments. Scarcity, then, serves as a catalyst for hardening the boundaries between national insiders and outsiders. Of equal importance is the role of scarcity as a narrative tool in justifying the exclusion of the other from the protection of the state (Carastathis, 2015). That is, the narrative of scarcity convinces individuals that there are not enough state resources to go around and that the solution to this issue is to exclude those who are “not deserving” enough—the national Others. For this reason, many scholars argue that a strong welfare state will work to dampen support for the radical right, both by alleviating economic hardship and by reducing the perception of competition over state resources (Swank and Betz, 2003).

### The Radical Right in CEE

The eight countries under consideration here—Bulgaria, Croatia, Czechia, Hungary, Poland, Slovakia, Slovenia, and Romania—share a common history of communist authoritarianism. All were on the Soviet side of the Iron Curtain, without being part of the USSR, thus maintaining some level of political autonomy. These post-communist nations thus share a common legacy of Communist rule, a system defined by “communist indoctrination, a planned and state-owned economy, a *de facto* (and often formally) one-party systems, and a class system that essentially lacked a bourgeoisie (Holmes, 2013, p. 1165). Furthermore, all eight are relatively recent admits to the EU: Czechia, Hungary, Poland, Slovakia, and Slovenia joined in 2004; Bulgaria and Romania in 2007; and, finally, Croatia in 2013 (Europa, 2019).

Following the collapse of the Soviet Union in the early 1990s, most of the CEE nations, as they democratized, underwent significant liberalization of their economies United as they are by populism and xenophobia, the radical right is not characterized by any particular economic position. Unlike more traditional conservative parties, at least part of the radical right has come out against neoliberalism, embracing (if only in rhetoric) socialist or populist economic positions—this has meant that, in the post-communist era, labor has turned right in certain CEE nations (Marinos, 2015; Ost, 2018). The Polish radical right, and the Law and Justice party in particular, has run on a platform of nationalist populism, campaigning against neoliberal economic reforms and global capitalism; this position has played at least some part in their popularity with the working class in Poland (Ost, 2018). Bulgaria ranks among the nations with the lowest quality of life in the EU (Kesy et al., 2015); given the economic and social hardships faced there, support for Ataka is perhaps not entirely surprising as the party has been campaigning for the renationalization of industry and the de-privatization of state services (Ghodsee, 2008). Thus, rather than representing a neoliberal position, the radical right in parts of CEE at least rhetorically represents itself as the solution to economic hardship and state retrenchment.

Scholars have also pointed to the ways in which globalization, capitalist expansion, and Europeanization disrupt traditional identities and values (Swindal, 2011). Neoliberalism is a favorite target, often tied to multiculturalism, “Islamicization,” and the weakening of the nation (Marinos, 2015). Beyond that, work on the Hungarian and Slovakian radical right has pointed to how mythical narratives of the nation, deployed by radical right parties, serve to shore up support for exclusionary and authoritarian politics (Pyltas, 2013). These narratives of the nation, and the constructions of identity they entail, are deeply informed by the communist past of the region. While some scholars have understood the vein of authoritarianism in CEE as a legacy of the Soviet era; conversely, others have argued that memories of communism as a foreign phenomenon forced on the nation from outside contribute to nationalist fears of loss of community and sovereignty (Korycki, 2019). These fears are mirrored in the Eurosceptic defenses of national interest and identity put out by the radical right (Hanley, 2004). In fact, drawing on anti-communist sentiment and alluding to memories of the communist era have served the radical right in parts of CEE particularly well, working as a source of political legitimacy (Swindal, 2011).

Despite no longer being under communist authoritarian rule, these nations remain politically unstable. In fact, scholars have suggested that the post-communist states are still states in transition, pointing to political crises, rampant corruption, and the rise of nationalist politics as signals that these nations have not fully transitioned to stable, liberal democracies (Holmes, 2013). All countries studied here implemented austerity measures after the financial crises in an attempt to follow EU guidelines on national debt and deficit (Pietras, 2009). Given their recent history of democracy engagement and EU involvement, the democratic backsliding and the rise of radical right politics in these nations are especially alarming.

As Table 1 shows, radical right parties have flourished across post-communist CEE, doing especially well in Hungary, Bulgaria, Poland, and Slovakia. Conversely, no significant radical right party has emerged on the political scene in Croatia or Romania, and the radical right parties in Czechia and Slovenia remain comparatively weak. The parties in the Table 1, diverse as they are, all fall under the umbrella categories of right-wing populism and radical right politics. Thus, they promote anti-immigrant and “law and order” politics, as well as adhere to an ethnic definition of the nation (Nordsieck, 2019). Bulgaria has a particularly varied radical right presence, with the number of radical right parties increasing in the years since the crash; that said, the level of support has remained fairly stable. The high levels of support for Jobbik in Hungary, averaging around 19% since the financial crisis, are particularly concerning, especially given the broader shift toward antidemocratic politics in the country. Victor Orbán, the Hungarian Prime Minister, and leader of the Fidesz party, has been gradually chipping away at the foundations of democracy in Hungary for several years, including undermining freedom of the press (Beauchamp, 2018).

**Table 1:** Electoral outcomes for radical right parties in the post-Iron Curtain countries.

	**Year**	**RR support**	**Parties**	**Mean post-2008**
Bulgaria	2009	9.4 + 4.1	Attack; Order Lawfulness Justice	13.25
	2013	3.7 + 7.3 + 1.9	National Front for the Salvation of Bulgaria (NFSB); Attack; Bulgarian National Movement (BNM)	
	2014	7.3 + 4.5 + 5.7	Coalition (NFSB and BNM); Attack; Bulgaria without Censorship	
	2017	9.1	Coalition of Attack, National Front for the Salvation of Bulgaria, Bulgarian National Movement	
Croatia	2011, 2015, 2016	–	–	–
Czechia	2010	–	–	7.25
	2013	6.9	Dawn	
	2017	10.6	Freedom and Direct Democracy	
Hungary	2010	16.7	Jobbik	18.67
	2014	20.2	Jobbik	
	2018	19.1	Jobbik	
Poland	2011	–	–	8.8
	2015	8.8	Kukiz'15	
Romania	2008, 2012, 2016	–	–	–
Slovakia	2010	5.1	Slovak National Party	8.83
	2012	4.6 + 1.6	Slovak National Party; People's Party Our Slovakia	
	2016	8.6 + 6.6	Slovak National Party; People's Party Our Slovakia	
Slovenia	2008	5.4	Slovenian National Party	3.4
	2011	1.8	Slovenian National Party	
	2014	2.2	Slovenian National Party	
	2018	4.2	Slovenian National Party	

*Nordsieck (2019)*.

## Analysis

Boolean analysis is a qualitative case comparison method, based largely on the logic of necessary and sufficient conditions. The power of this method of analysis comes from its use in examining different pathways leading to the same outcome. In Boolean analysis, causal conditions are coded into binary variables: 1 where a condition is present or strong and 0 where it is absent or weak. In this way, the truth table assembled shows the pathways possible among the comparison countries, allowing for an assessment of the necessary or sufficient conditions (Veugelers and Magnan, 2005). To this end, the case study countries were sorted by outcome—that is, by the strength of weakness of radical right party electoral success. Using the data on electoral outcomes, countries were sorted by the median of the mean level of electoral support in national-level elections in the years since the crash, thus providing a measure of where, in the CEE nations studied, the radical right is stronger or weaker (see Table 1) (Nordsieck, 2019).

Next, four causal variables were included in the analysis. Given the importance placed on the strength of the welfare state in decreasing support for the radical right through dampening perceived economic hardship, a measure for welfare effort was included in the analysis. Welfare effort was measured as the percentage of the country's gross domestic product (GDP) spent on social protection and benefits (Eurostat, 2019c). Like the outcome variable of radical right party success, this variable was coded using the median of the means of social protection expenditure from 2008 to 2017, providing a measure for the relative strength of the national welfare effort (see Appendix 1). In the eight countries analyzed, the average expenditure on social protection between 2008 and 2017 ranged from 12.09 in Romania to 16.17 in Hungary.

Next, in an effort to measure the role of economic grievances, a condition for comparatively high unemployment was included. Using data from the (World Bank: Bulgaria, n.d.; World Bank: Hungary, n.d.; World Bank: Poland, n.d.; World Bank: Romani, n.d.; World Bank: Slovakia, n.d.; World Bank: Slovenia, n.d.; World Bank: Croatia, n.d.; World Bank: Czech Republic, n.d.) high unemployment was coded using the median of the mean levels of unemployment in each country from 2008 to 2017. Czechia reported the lowest average for unemployment from 2008 to 2017, at 5.70%, while the highest rates of unemployment in the last decade were found in Croatia, where the average was 13.40% (see Appendix 2).

Finally, several population measures were included to assess the effect of emigration and immigration on support for the radical right. First, using data from (Eurostat, 2018), countries were sorted on the basis of whether or not their inter-EU emigration had increased over the past decade (see Figure 1). Thus, I coded as 1 the countries where the number of citizens living and working in another EU country had increased between 2007 and 2017 and as 0 the countries where it had fallen or stayed stable. Only Croatia, Czechia, and Slovenia were coded as 0, while Bulgaria, Hungary, Poland, Slovakia, and Romania were coded as 1.

A measure for high immigration was also included in the analysis—that is, for the percentage of foreign-born residents in each nation, averaged across the last decade (where data as available). Eurostat considers those nations where more than 10% of the population is foreign born to have a high proportion of immigrants (Eurostat, 2019a). This same measure was used here, so that those countries were over 10% of the population was foreign were coded as 1, for high immigration; conversely, those countries where immigrants made up <10% of the population were coded as 0. Only Croatia and Slovenia were coded as 1—in all other six countries, the percentage of the population that was foreign born averaged <5% over the last decade (see Appendix 3).

This is, obviously, a limited selection of the possible independent variables associated with support for nationalist politics identified in the literature. Indeed, researchers working on the radical right have explored a range of structural and societal factors shown to predict radical right party success such as a polarized political system, weak conservative parties, the use of proportional representation, and high levels of inequality (Carter, 2002; Bale, 2003; Arzheimer and Carter, 2006; Han, 2016). Similarly, sociodemographic factors—such as gender, education level, and occupational sector—have also been shown to be powerful predictors of individual support for radical right parties (Coffé, 2018). There is considerable debate in the literature about the exact nature of the relationship between economic hardship (often measured through unemployment) and migration in driving radical right party success, and this work aims to respond to these debates (Golder, 2003; Rydgren, 2007, 2008; Halla et al., 2017). Given how much of this work focuses on Western Europe, and the particular economic and migratory context there, it remains an open question as to how generalizable the arguments for high immigration and high unemployment are for explaining the rise of the radical right in the rest of Europe, as well as in the CEE nations. Furthermore, given the nature of the methods used in the analysis presented here, the more subjective side of migration and economics—that is, the experience of it—is overlooked. Radical right-wing attitudes, characterized by authoritarian leanings and anti-immigrant sentiments, have been investigated at the aggregate level and have been theoretically tied to real and perceived scarcity, hardening of national boundaries, and a disengagement with mainstream politics (Carastathis, 2015; Hjorth, 2016; Lamprianou and Ellinas, 2017). However, in order to focus in on the economic structural factors driving support for radical right politics and in order to keep the focus of the research narrow enough to be feasible, I have excluded political structural and sociodemographic variables from my analysis. Thus, in an attempt to situate myself in the debate over the roles of migration and economic hardship within this literature, I am focusing on the variables of welfare effort, unemployment, immigration, and emigration.

As shown in the Table 2, the most common pathway to a stronger radical right presence was high national employment coupled with increasing emigration, irrespective of the strength of the welfare effort. Additionally, while none of the nations with strong radical right have high immigration, high immigration is also not necessarily related to a weak radical right presence. Thus, the most straightforward pathway to a strong radical right presence in the comparison countries is as follows:

*RR* = *WE UE IE hi* + *we UE IE hi*

*or put simply*,

*RR* = *UE IE*

The sole exception to this rule is Poland, where a strong radical right presence has emerged in a context of increased emigration, low immigration, low unemployment, and a strong welfare effort. Thus, increased emigration appears to be necessary, but not sufficient, to the presence of a strong radical right group, explaining its variant relationship with the outcome variable, that of the strength of the radical right presence. As the analysis shows, with the exception of Poland, increased emigration works with high unemployment to produce conditions amenable to the radical right. This is further demonstrated by those nations where the radical right is either weak or absent: none of those countries categorized as having a weak radical right presence present with both high unemployment and increased emigration. Again, the strength of the welfare effort seems to have little determinant effect. Rather, absence of either increased emigration or high unemployment, or of both conditions, appears to significantly weaken the radical right. Thus, the key pathway for radical right electoral strength, at least in the CEE countries, is increased emigration co-occurring with comparatively high rates of unemployment.

**Table 2:** Boolean analysis of radical right electoral presence.

		**Welfare effort (WE)**	**High unemployment (UE)**	**Increased emigration**	**High immigration (HI)**
Stronger radical right electoral presence	Bulgaria	0	1	1	0
	Hungary	1	1	1	0
	Poland	1	0	1	0
	Slovakia	0	1	1	0
Weaker radical right electoral presence	Croatia	1	1	0	1
	Czechia	0	0	0	0
	Romania	0	0	1	0
	Slovenia	1	0	0	1

## Discussion

The recent electoral success of radical right parties in post-communist CEE poses a pressing problem for the future of democratic politics in the region. Traditional approaches to understanding the impetus driving antidemocratic politics in Europe tend to focus on Western Europe. These approaches underline the detrimental effects of high unemployment and high immigration on prodemocratic political attitudes (Golder, 2003). However, post-communist CEE does not fit this picture—despite the significant increases in population movement over the past decade or so, accelerated by accession into the EU, CEE countries continue to see significantly less immigration than other parts of Europe; in fact, they have some of the lowest rates of foreign-born residents in Europe (Eurostat, 2019d). Although these countries were adversely impacted by the financial crisis in 2008, their unemployment rates remain low when compared to those in certain Western European nations, notably Greece and Spain. Furthermore, the presence of a non-strict proportional representation system, where the threshold for party status is under 4% (Veugelers and Magnan, 2005), has been associated with the electoral success of radical right parties. However, the lack of variation between the case countries in electoral systems meant that this variable was of little analytic use: all except Hungary, which uses mixed-member proportional, had a strict proportional representation system in place (Nordsieck, 2019). Thus, traditional structural explanations, ones which center on immigration and unemployment levels, fail to adequately explain the rise of radical right parties in this context.

A more analytically productive approach to understanding the rise of electoral support for the radical right comes by considering perceptions of hardship, as well as economic grievance, rather than objective levels of unemployment and immigration. The analysis, presented above, showed that the key pathway for electoral success of the radical right in the comparison countries was through a co-occurrence of increased emigration and comparatively high unemployment. While it is true that emigration rates are still generally higher among Western European countries, the rates of emigration have increased exclusively in CEE since the financial crisis (Eurostat, 2019d). High unemployment undoubtedly plays some causal role in the increasing rate of emigration (Waterbury, 2018); however, it also points to a poor economic situation in the home country. In this way, unemployment rates shape the trajectories of both those who emigrate and those who stay.

Austerity politics, as mentioned earlier, have both a material component and an ideological component. First, austerity policies result in real cutbacks to social benefits and protections, significantly impacting quality of life within a nation state (Pietras, 2009; Juska and Woolfson, 2015). This decrease in quality of life, then, pushes those in more precarious labor situations—often the youngest—out as they search for better economic opportunities (Juska and Woolfson, 2015; Waterbury, 2018). Second, at a more abstract level, these cuts promote perceptions of scarcity of state resources. Perceptions of scarcity work simultaneously to stoke fears of misuse of state resources by those deemed undeserving and to discursively justify the exclusion of national outsiders from the wealth and protection of the state (Carastathis, 2015). Just as perceptions of scarcity are driven by the cuts to welfare protections, so too are they amplified by the rhetoric of fiscal severity and restriction of austerity politics. Thus, the perception of scarcity comes to serve as a justifying narrative for the exclusion of national others from access to the “scarce” resources of the state, further serving to harden the boundaries between national insider and outsider (Carastathis, 2015). Interestingly, in the analysis presented above, the welfare effort of the state appeared to matter little in determining the strength of electoral support for the radical right. This finding gives credence to the argument that objective structural conditions matter much less for support for the radical right than to perceptions of hardship and the attendant loss of trust in state and civil institutions (Lamprianou and Ellinas, 2017).

As mentioned earlier, the key pathway to a strong radical right was increased emigration co-occurring with comparatively high rates of unemployment. Poland, however, was a notable exception to this trend, having a relatively strong far right presence, without the attendant high unemployment rates. Rather than discounting the effects of unemployment on radical right support, the Polish case points to nuance in the role of unemployment; it may well be that the experience of the labor market as difficult and scarce is a more important factor in driving radical right sentiment than the actual unemployment rate in a country. Poland, having not taken up the Euro upon EU accession, suffered less than some other CEE nations after the financial collapse of 2008. However, years of neoliberal labor and economic reforms did mean that Poland had become a European leader in part-time work, wage stagnation, and unequal development by the end of the first decade of this century (Ost, 2018). Thus, although Polish unemployment has remained low, much of the labor force is trapped in precarious work or has resorted to emigrating for better opportunities (Ost, 2018). Within this context, Law, and Justice—the leading far-right party in Poland—has taken up a nationalist populist economic agenda, vowing to help and protect the working classes from the ravages of global capitalism (Ost, 2018). In this way, despite comparatively low rates of unemployment, the precarious state of the Polish labor market may be driving radical right support.

The large-scale emigration being experienced by certain Central and Eastern European states has also been perceived as a threat to the cultural and physical survival of the nation (Waterbury, 2018). Historically, migration has played a significant role in nation-building. Control over borders is, of course, of utmost importance to nation-state sovereignty, but so is control over the passage of people over them. When the nation comes to be ethnically defined, the regulation of co-ethnic movement serves an important role in consolidating the nation. This was the case, as shown by Brunnbauer (2012) in Yugoslavia in the interwar years. The state, then, pursued an ethnically differentiated emigration policy, where the emigration of ethnic Slavs was strictly controlled, while the emigration of non-ethnic Slavs was encouraged (Brunnbauer, 2012). This case shows the importance attributed to territory in the definition of national identity. The high importance placed on territory to identity sheds light on the pervasiveness of xenophobia in Eastern Europe (Ceobanu and Escandell, 2008): the encroachment of Others onto national territory becomes more threatening when control over territory is important to identity.

Nations vary both in the content of their national identification and in the degree to which national identity is exclusionary. Moreover, the analysis conducted here suggests a relationship between increasingly exclusionary conceptions of national identity and austerity politics. Thus, austerity politics and economic hardship provide the material and ideological conditions under which emigration comes to be seen as a threat to the well-being of the nation. Specifically, austerity politics drive support for radical right politics in three principal ways. First, austerity-driven cuts to social protections and benefits weaken trust in civic institutions, driving anti-immigrant, and antiestablishment politics. Next, welfare chauvinism, borne from perceptions of scarcity, hardens the lines between national insiders and outsiders, prompting support for the exclusion of national outsiders from the protection of the state. Finally, as increasing numbers of nationals depart for better economic opportunities, the linking of territory to ethnicity to nationality comes under increasing pressure. This adds to the appeal of parties—generally found on the radical right—who advocate for an increased emphasis on the ethnic roots of national identity. In this way, large-scale emigration, within the context of an open-borders continent, has spurred a discursive hardening of the boundaries of national belonging in some political quarters.

## Conclusion

The years since the 2008 financial crisis have seen a concerning rise in antidemocratic politics across Europe, and post-communist CEE is no exception. Of the CEE eight countries studied here, Bulgaria, Hungary, Poland, and Slovakia have comparatively strong radical right parties. Of these, Hungary's Jobbik party is the strongest, garnering roughly 19% of the electoral vote in each election since 2008 (Nordsieck, 2019). These parties, like others on the radical right of the political spectrum, advocate politics of anti-immigrant xenophobia and an antiestablishment populism (Rydgren, 2007). Given the relatively recent democratic transition in these countries, this democratic backsliding, and the rise popularity of radical right parties, is of particular concern.

The analysis conducted for this paper suggests that, within a context of economic hardship and austerity, increased emigration is understood as a threat to the well-being of the nation, both economically and socially. Given the freedom of movement granted to EU/EFTA citizens, the increased emigration seen in this part of the world has spurred a de-linking of territory and ethnonationality, placing pressure on existing understandings of national identity. Coupled with the perceptions of scarcity brought about by the austerity measures put in place following the financial crises, emigration had come to be seen as a grave threat to the survival of the ethnonational insiders, thereby stoking support for radical right parties who advocate for a strengthening of the ethnic character of nationality and citizenship. Migration, then, does not only affect labor-receiving nations but also has deep impacts on the political character of labor-sending nations.

Understanding the differential effects of labor movement on the political atmosphere in labor-sending and labor-receiving countries is also of importance in the current political climate. There is a significant body of work focused on the political effects of mass immigration into Western Europe, showing how radical right parties stoke fears of immigrants, thereby building up support for their exclusionary politics (Golder, 2003; Cochrane and Nevitte, 2014; Careja et al., 2016; Hjorth, 2016). However, there is good reason to think that this relationship is not generalizable to the CEE context: as population statistics show, immigration remains low in CEE; statistics collected by the EU show that, in fact, immigration to many Eastern European states remains incredibly low, with foreign-born residents making up <2% of the population in Romania and Poland (Eurostat, 2019a,b). In fact, it appears that emigration is perceived as a larger threat than immigration in certain CEE nations. In an effort to situate myself within the debate on economic hardship and migration as conditions driving support for radical right parties, I restricted my analysis to a narrow range of variables and to eight CEE nations.

The issues of EU enlargement and economic hardship are also inexorably linked in postcrash Europe, in particular when it comes to migration. Just as potential migrants from CEE are induced to leave their countries for economic opportunity, their movement around Europe—in particular, into Western Europe—is made easier by the entrance of the CEE countries into the EU and by the freedom of movement guaranteed to EU citizens (Eurostat, 2018; Waterbury, 2018). Furthermore, there is a strong strain of Euroscepticism among the European radical right, meaning that at least some of the growth in radical right support is driven by anti-EU sentiment and fears about loss of sovereignty due to EU enlargement (Kentmen-Cin and Erisen, 2017). Finally, much of the austerity undertaken in CEE was at the behest of the EU and the IMF, further complicating the relationship between the EU and austerity in CEE (Santa, 2012; Cankar and Petkovsek, 2013; Walter, 2016).

There are particular limitations to the generalizability of this research, primarily due to the narrow scope. As mentioned briefly in the analysis, previous research on the European radical right has examined factors ranging from the political system structure to sociodemographic variables as predictors of radical right success (Golder, 2003; Rydgren, 2007, 2008; Halla et al., 2017). Given that the analysis carried out here was focused on the interaction between migration and economic hardship, there are likely a myriad of other factors at play—structurally, socially, and individually—influencing support for radical right and nationalist politics in the CEE region. Moreover, there is a lack of intranational comparative work on radical right politics; future work, then, could examine in more detail the variation in support within nations, narrowing in on what kinds of people support nationalist and radical right politics in what contexts. A more qualitative approach may prove useful here in examining the kinds of rhetoric employed by parties, as well as how it may appeal to particular segments of society. Finally, given the findings here about the role of emigration in driving radical right support, future work concerning the debate of migration and economic hardship could compare Western and Eastern Europe, examining the relationship between nationalism and labor movement through a more comparative lens.

## Data Availability Statement

All datasets generated for this will be included in the manuscript/Supplementary Files.

## Author Contributions

MF undertook the analysis and writing and responsible for the framing, editing, and direction of the paper.

### Conflict of Interest

The author declares that the research was conducted in the absence of any commercial or financial relationships that could be construed as a potential conflict of interest.
