# Supplementary material for: The Costs of Austerity: Labor Emigration and the Rise of Radical Right Politics in Central and Eastern Europe
Source: Front Sociol. 2019 Oct 11;4:69. doi: 10.3389/fsoc.2019.00069 (PMC8022759; doi:10.3389/fsoc.2019.00069)
Supplement: Supplementary file 1 [file Presentation_1.pdf]

Appendices

Appendix 1. Welfare effort of the case study countries, 2008 to 2017.

	2008	2009	2010	2011	2012	2013	2014	2015	2016	2017	Mean
**Bulgaria**	10.7	12.9	12.9	12.2	12.4	13.5	13.4	13.3	12.7	12.5	**12.65**
**Croatia**	13.4	15.0	14.9	15.3	15.1	15.2	15.6	15.7	14.6	14.3	**14.91**
**Czechia**	11.9	13.1	13.2	13.2	13.3	13.5	13.1	12.5	12.3	12.0	**12.81**
**Hungary**	17.4	18.1	17.4	16.9	16.7	16.5	15.4	14.8	14.5	14.0	**16.17**
**Poland**	15.5	16.1	16.3	15.5	15.6	16.0	15.9	15.7	16.6	16.4	**15.96**
**Romania**	11.0	13.1	13.9	13.0	12.4	11.5	11.4	11.4	11.5	11.7	**12.09**
**Slovakia**	12.6	15.1	15.3	14.7	15.0	15.3	15.1	15.0	15.1	14.5	**14.77**
**Slovenia**	15.5	17.5	18.1	18.7	18.5	18.6	17.8	17.3	16.8	16.2	**17.5**
**Calculated as the percentage of GDP spent on social protections and benefits by the state<br>Source: Eurostat 2019**											

Appendix 2. Unemployment rates in the case study countries, 2008 to 2017.

	**2008**	**2009**	**2010**	**2011**	**2012**	**2013**	**2014**	**2015**	**2016**	**2017**	**Mean**
**Bulgaria**	5.61	6.82	10.28	11.26	12.27	12.94	11.42	9.14	7.57	6.16	**9.347**
**Czech Republic**	4.39	6.66	7.28	6.71	6.98	6.95	6.11	5.05	3.95	2.89	**5.697**
**Croatia**	8.53	9.2	11.62	13.68	15.93	17.25	17.29	16.18	13.1	11.21	**13.399**
**Hungary**	7.82	10.03	11.17	11.03	11	10.18	7.73	6.81	5.11	4.16	**8.504**
**Poland**	7.12	8.17	9.64	9.63	10.09	10.33	8.99	7.5	6.16	4.89	**8.252**
**Romania**	5.79	6.86	6.96	7.18	6.79	7.1	6.8	6.81	5.9	4.93	**6.512**
**Slovenia**	4.37	5.86	7.24	8.17	8.84	10.1	9.67	8.96	8	6.56	**7.777**
**Slovakia**	9.51	12.03	14.38	13.62	13.96	14.22	13.18	11.48	9.67	8.13	**12.018**
**Source: World Bank, n.d.**											

<u>Appendix 3. Foreign-born as a percentage of the national population, 2009 to 2018</u>

		2009	2010	2011	2012	2013	2014	2015	2016	2017	2018	Average
**Bulgaria**	**Total**			7,369,431	7,327,224	7,284,552	7,245,677	7,202,198	7,153,784	7,101,859	7,050,034	
	**Foreign-born**			78,621	85,076	96,113	109,239	123,803	136,421	145,390	156,505	
	**Percentage**			1.067	1.161	1.319	1.508	1.719	1.907	2.047	2.220	**1.619**
**Croatia**	**Total**					4,262,140	4,246,809	4,225,316	4,190,669	4,154,213	4,105,493	
	**Foreign-born**					574,383	568,678	561,093	547,929	539,588	528,982	
	**Percentage**					13.476	13.391	13.279	13.075	12.989	12.885	**13.183**
**Czechia**	**Total**	10467542	10506813	10532770	10505445	10516125	10512419	10538275	10553843	10578820	10610055	
	**Foreign-born**	384,161	398,493	387,971	390,843	387,337	396,156	416,454	433,290	465,056	467,580	
	**Percentage**	3.670	3.793	3.683	3.720	3.683	3.768	3.952	4.106	4.396	4.407	**3.918**
**Hungary**	**Total**	10030975	10014324	9,985,722	9,931,925	9,908,798	9,877,365	9,855,571	9,830,485	9,797,561	9,778,371	
	**Foreign-born**	424,031	436,616	443,295	401,769	423,317	447,029	475,508	503,787	513,649	536,182	
	**Percentage**	4.227	4.360	4.439	4.045	4.272	4.526	4.825	5.125	5.243	5.483	**4.655**
**Poland**	**Total**	38135876	38022869	38062718	38063792	38062535	38017856	38005614	37967209	37972964	37976687	
	**Foreign-born**	453,248	642,417	637,408	630,478	625,363	620,308	611,855	626,396	651,845	695,850	
	**Percentage**	1.189	1.690	1.675	1.656	1.643	1.632	1.610	1.650	1.717	1.832	**1.629**
**Romania**	**Total**					20020074	19947311	19870647	19760585	19644350	19530631	
	**Foreign-born**					182,939	211,210	281,048	347,344	421,801	508,625	
	**Percentage**					0.914	1.059	1.414	1.758	2.147	2.604	**1.649**
**Slovakia**	**Total**		5,424,925	5,435,273	5,404,322	5,410,836	5,415,949	5,421,349	5,426,252	5,435,343	5,443,120	
	**Foreign-born**		64,422	68,135	156,883	158,164	174,908	177,624	181,642	186,217	190,308	
	**Percentage**		1.188	1.254	2.903	2.923	3.229	3.276	3.347	3.426	3.496	**3.283**
**Slovenia**	**Total**	2,032,362	2,046,976	2,050,189	2,055,496	2,058,821	2,061,085	2,062,874	2,064,188	2,065,895	2,066,880	
	**Foreign-born**	243,404	253,786	228,588	230,109	232,703	235,310	237,616	241,203	245,369	250,226	
	**Percentage**	11.976	12.398	11.150	11.195	11.303	11.417	11.519	11.685	11.877	12.106	**11.663**
**Source: Eurostat 2019**												
